# Metabolic Outcomes in Pediatric Patients One-Year Post-Total Pancreatectomy with Islet Autotransplantation after Early Pump Initiation

**DOI:** 10.3390/jcm12093319

**Published:** 2023-05-06

**Authors:** Siobhan Tellez, Lindsey Hornung, Maisam Abu-El-Haija, Deborah Elder

**Affiliations:** 1 Division of Endocrinology, Cincinnati Children’s Hospital, Cincinnati, OH 45229, USA; 2 Division of Biostatistics and Epidemiology, Cincinnati Children’s Hospital, Cincinnati, OH 45229, USA; 3 Division of Gastroenterology, Cincinnati Children’s Hospital, Cincinnati, OH 45229, USA; 4 Department of Pediatrics, College of Medicine, University of Cincinnati, Cincinnati, OH 45229, USA

**Keywords:** TPIAT, islet autotransplantation, pediatrics

## Abstract

We previously published that insulin pump initiation immediately after IV insulin therapy was associated with improved post-surgical glycemic outcomes compared to multiple daily injections (MDI) in pediatric patients following a total pancreatectomy with islet autotransplantation (TPIAT). We investigated metabolic outcomes of this population at one-year post-TPIAT to assess if the improved outcomes in the early pump group were sustained over time. We retrospectively reviewed 40 patients post-TPIAT previously studied at 10-days post-surgery (15 used MDI and 25 used pump therapy immediately post-ICU, and all were discharged on pump therapy). Data analyzed included: demographics, islet equivalents per kilogram (IEQ/kg) transplanted, exogenous insulin use, and baseline vs. one-year (via mixed meal testing) HbA1c, fasting glucose, insulinogenic index, and the area under the curve (AUC) for insulin and c-peptide. More patients were off insulin at one year in the early pump group compared to the MDI group (45% vs. 13%, *p* = 0.07). Of all patients off insulin, 100% of the early pump users weaned off by six months post-TPIAT compared to 30% of the MDI users. Two known variables associated with favorable insulin outcomes, lower age and higher IEQ/kg, were not significantly different between groups. Fasting glucose was lower in the early pump group compared to the MDI group (median 97 vs. 122 mg/dL, *p* = 0.003), while AUC c-peptide was greater in early pump users at one-year post-TPIAT but did not reach significance (median 57.0 vs. 50.3 ng/mL × minutes, *p* = 0.14). Other metabolic outcomes did not differ between groups. Despite lower median age and higher IEQ/kg in the MDI group, the early pump group had a lower fasting glucose. Younger TPIAT age (*p* = 0.02) and early pump users (*p* = 0.04) were significantly associated with insulin independence at one year. This study was limited by sample size. Early pump use may have long-term benefits in post-TPIAT endogenous insulin secretion.

## 1. Introduction

Total pancreatectomy with islet autotransplantation (TPIAT) is a surgical treatment approach for individuals with severe chronic pancreatitis (CP) or acute recurrent pancreatitis (ARP) that have exhausted other medical and endoscopic treatment options. The autologous transplant of islet cells from the pancreas, with subsequent maintenance of optimal conditions for islet engraftment, is aimed at potentially (or partially) mitigating insulin-dependent post-pancreatectomy diabetes mellitus [1,2,3]. Some predictors of insulin independence in pediatric TPIAT recipients include an islet yield at the time of surgery (IEQ/kg) > 5000 and younger age at surgery [4]. After TPIAT, the literature suggests that the maintenance of blood glucose in the range of 80–120 mg/dL during the islet engraftment period promotes optimal outcomes [1,5]. However, there remains a need to expand knowledge on best practices in glycemic management post-TPIAT to consistently and safely achieve the strict glycemic target range and promote optimal longer-term outcomes. 

We previously published findings on the improved glycemic outcomes of pediatric post-TPIAT patients who transitioned from IV insulin directly to insulin pump therapy during the first 10-days post-ICU compared to those who were managed with multiple daily injections (MDI) and then transitioned to pump use [6]. We hypothesized that not only would using insulin pump therapy for post-TPIAT glycemic management improve glycemic outcomes in the critical immediate post-operative period, but those benefits would extend to longer-term metabolic outcomes. By investigating metabolic outcomes of subjects at one-year post-TPIAT, this paper aims to assess if the improved outcomes of the early pump initiation group were sustained over longer time periods.

## 2. Materials and Methods

A retrospective review of patients post-TPIAT who were previously studied at 10-days post-surgery were reviewed at 12-months post-TPIAT. This cohort of patients included individuals who were managed on MDI within the first 10-days post-ICU before transitioning to an insulin pump, and individuals who were managed on insulin pumps directly post-ICU [6]. In the early years of the program, all TPIAT recipients were transitioned from IV insulin in the ICU to MDI while they recovered on a surgical unit, and then transitioned from MDI to an insulin pump while they completed post-surgical recovery and education on an endocrine unit [6]. Programmatic advancement in 2016 allowed for all later TPIAT recipients to transition from IV insulin directly to an insulin pump on the endocrine unit, omitting the use of MDI and the transfer to and from a surgical unit during hospitalization [6]. All TPIAT recipients since the start of the program used continuous glucose monitoring during hospitalization. All TPIAT recipients were discharged from hospital on an insulin pump and a continuous glucose monitor.

The islet isolation procedure at our center has been previously described [7]. Islet infusion into the portal venous system occurs via a short splenic vein stump while systemically heparinizing the patient to reduce the risk of thrombosis. Islet isolation from the pancreatic tissue uses a semi-automated Ricordi method for separation [7]. The islet yield is estimated after the final wash.

Data analyzed included: demographic information, IEQ/kg transplanted at time of surgery, exogenous insulin use at 1 year (on vs. off), and baseline vs. 1-year measures (via mixed meal tolerance testing (MMTT)) of HbA1C, fasting glucose, insulinogenic index, c-peptide secretion index, and area under the curve (AUC) for insulin and c-peptide. Functional outcomes of the islet grafts were assessed using Igls 2.0 criteria and BETA-2 score [8,9,10]. The insulinogenic index is commonly used to estimate beta cell function in the first phase of insulin secretion and is calculated from an oral glucose tolerance test (OGTT) or mixed meal tolerance test [11]. The AUC is a standard tool for assessing the secretory potential of beta cells after stimulus [12] and was calculated based on the midpoint of each interval for times 0, 30, 60, 90, and 120 min using Riemann sums. The c-peptide secretion index is the ratio of postprandial c-peptide to glucose, a validated assessment of beta cell function [13]. Igls 2.0 criteria uses HbA1c, c-peptide levels, severe hypoglycemic events, and insulin requirement to categorize beta cell function as “optimal”, “good”, “marginal”, or “failure” [8]. BETA-2 uses fasting glucose, c-peptide, HbA1c, and the total daily insulin dose per kilogram to score beta cell function [9,10]. While Igls 2.0 criteria provides some standardization to the assessment of graft function, use of BETA-2 scoring for pediatric TPIAT recipients to assess post-surgical beta cell function is increasing in the literature and was included in this analysis [4]. Baseline data were defined as the metabolic measures collected within 1 year prior to their TPIAT. Patients who required insulin at 1 year received basal insulin coverage only during MMTT to minimize impact on interpretation of insulin levels.

MMTT was scheduled with a start time between 0800 and 1100 for all patients, depending on timing and scheduling of other multi-disciplinary appointments. Patients were instructed to fast for 8 h prior to MMTT. At the start of the test, patients received 6 mL/kg (maximum of 360 mL) of Boost^®^ (Société des Produits Nestlé S.A., Vevey, Switzerland) high-protein nutritional drink (carbohydrate content 41 g per mL). Serum blood samples via venipuncture were drawn at 30 min intervals until 120 min post-ingestion. At each interval, the stimulated glucose, insulin, and c-peptide profiles were collected. Baseline MMTTs occurred within the 12 months prior to TPIAT.

This study was approved by the Institutional Review Board (2019-0608). Informed consent and assent to participate in the Pancreas Care Center clinical registry were obtained from parents and patients. Subsets of this population have been included in prior publications through studies with different designs from the current study [4,14,15,16].

Data were analyzed using SAS^®^ version 9.4 (SAS Institute, Cary, NC, USA). Due to skewed distributions and small numbers, continuous data were summarized as medians with interquartile ranges (IQR: 25th–75th percentiles), while categorical data were summarized as frequency counts and percentages. For continuous data, non-parametric Wilcoxon–Mann–Whitney tests were used to compare characteristics between groups. For within-group paired comparisons for BMI percentile, Wilcoxon signed-rank tests were used. Chi-square or Fisher’s exact tests were used, as appropriate, for group comparisons of categorical data. Logistical regression models were used to examine insulin outcomes between groups while controlling for other covariates. A *p*-value < 0.05 was considered statistically significant.

## 3. Results

A total of 40 patients post-TPIAT, who were previously studied at 10-days post-surgery (15 managed with MDI and 25 managed with pump therapy immediately post-ICU), were reviewed at 12-months post-TPIAT. The MDI users had a median ICU duration of 10 days (IQR: 9.0–12.0) compared with a median ICU duration of 7.0 (IQR: 7.0–8.0) days in early pump users (*p* < 0.0001) (Table 1). The median IV insulin treatment duration for MDI users was 9.0 (IQR: 8.0–11.0) days compared with median IV insulin treatment duration of 7.0 (IQR: 7.0–8.0) days (*p* = 0.001).

The median time MDI users were on injections before transitioning to pumps was five days (IQR: 4.0–9.0). The length of stay on the endocrine unit was equivalent between the two groups, but given the differences in the median length in the ICU (7.0 for early pump users vs. 10.0 for MDI users) and the additional time on the surgical unit for the MDI users (median of five days), the early pump users had a significantly shorter median total length post-ICU compared to MDI users (11.5 days vs. 16.5 days, *p* = 0.005) [6].

There were four patients where a pre-TPIAT MMTT could not be performed due to scheduling limitations and only fasting blood samples were collected. At 12-months post-TPIAT, six patients did not have MMTT results due to missed appointments or being lost to follow-up. Even for patients who completed MMTT as expected, there were some missing data points due to hemolysis or collection error. Due to the potential of underestimating beta cell function, only complete MMTTs were included in analysis.

There were no differences between groups with regards to demographic characteristics such as age, IEQ/kg transplanted at surgery, or etiology of pancreatitis (Table 2). The groups did not significantly differ at baseline or at 12 months for BMI percentile (Table 3 and Table 4). The BMI percentile for MDI users did not significantly differ between baseline and 12 months (*p* = 0.52); however, the BMI percentile for early pump users significantly decreased (*p* = 0.003).

There were no differences in baseline HbA1c, fasting glucose, or c-peptide (Table 3).

At one-year post-TPIAT, the fasting glucose was lower in the early pump group compared to the MDI group (97 vs. 122 mg/dL, *p* = 0.003) (Table 4). The AUC for c-peptide was greater in early pump users at one-year post-TPIAT, though the p-value was not significant (*p* = 0.14). The other indices of beta cell function did not differ between groups.

A modified version of the Igls 2.0 criteria was used as some of the patients did not fit within the groupings (Table 5). At one-year post-TPIAT, more of the early pump users had optimal beta cell graft function (10/21, 48%) compared with the MDI users (2/14, 14%). The BETA-2 score for MDI users was lower at 8.0 (5.1–11.5) compared with the early pump users at 13.7 (10.7–16.5), though the p-value was not significant (*p* = 0.06) (Table 6).

At one-year post-TPIAT, 45% of early pump users (n = 10/22) were off insulin compared with 13% (n = 2/15) of MDI users, *p*-value (*p* = 0.07) (Table 7). After using a logistic regression model to simultaneously take other covariates into account, age at TPIAT (*p* = 0.02) and early pump use (*p* = 0.04) were significantly associated with insulin independence at one-year post-TPIAT while sex was not (*p* = 0.60). Of all individuals who were off insulin at one year, all of the early pump users came off of insulin within six months of surgery. The median HbA1c at one-year post-TPIAT for MDI users was 6.7% (5.9–7.8) compared with 6.2% (5.6–7.4) in the early pump users (*p* = 0.40). In the MDI user group, three of the individuals who were still on insulin had transitioned from pump therapy back to MDI and the remaining nine individuals still on insulin continued pump therapy. In the early pump user group, only one individual who was still on insulin had transitioned to MDI, and all other individuals requiring insulin continued on pump therapy.

## 4. Discussion

At one-year post-TPIAT, the early pump users had favorable glycemic outcomes compared to the MDI users in the following parameters: lower fasting glucose, greater stimulated c-peptide production during MMTT, and a higher proportion off insulin. Both groups had optimal management of diabetes despite differences in metabolic outcomes.

This study is novel since it is the first to examine longer-term metabolic differences in outcomes based on the approach to insulin management in the immediate post-operative period. Understanding insulin management approach options, and the potential impact on long-term metabolic outcomes, is a key component of promoting optimal post-TPIAT diabetes management.

Of the metabolic characteristics assessing beta cell function, the fasting glucose was the only outcome that reached statistical significance at one year. The insulinogenic index and c-peptide secretion index were not different between groups, and similarly suggested impaired beta cell function in both groups (an expected finding for transplanted islets). However, the AUC for c-peptide was higher in early pump users (though not statistically significant), which may demonstrate enhanced secretion from the transplanted beta cells later in the insulin-producing phase. Amongst the early pump users, 71% (15/21) of the patients had optimal or good beta cell graft function compared with 36% (5/14) of the MDI users. These data suggest a trend toward improved post-prandial glycemic control amongst the early pump users, who were also more likely to be off insulin. This is clinically relevant, as the long term clinical benefit of preserved beta cell function evidenced by stimulated c-peptide directly affects HbA1c and indirectly affects complications such as nephropathy and retinopathy [12]. Additionally, these findings are consistent with the findings in a paper by Swauger et al. [4], who reported that higher values of stimulated peak c-peptide after TPIAT correlated with lower insulin use (and therefore improved beta cell function) at one-year post-TPIAT.

More of the early pump users were insulin independent at 12-months post-TPIAT, which is the most ideal outcome of islet autotransplantation. Once age was taken into account, the early pump users were significantly more likely to be insulin independent. The improved beta cell function in the pump user group, along with the optimal mean HbA1c, indicate that even when patients require exogenous insulin, the early introduction of pump therapy may have influenced the retention of endogenous beta cell function.

Diabetes technology, currently approved and optimized for use in individuals with type 1 and type 2 diabetes, will be increasingly impactful on the long-term outcomes for individuals with post-pancreatectomy diabetes. There is growing literature on the long-term survival outcomes in adults post-TPIAT but less evidence on supporting optimal longitudinal outcomes for pediatric TPIAT recipients [17,18]. Amongst adults, Wilson et al. reports 10.9% deaths from diabetes-related causes and 12.7% deaths from cardiovascular causes [18], which underscores the importance of optimizing management of diabetes in a manner that the family and/or individual can sustain over a lifetime. CGM use is recognized as a critical component of achieving and sustaining glycemic targets in adults and children with type 1 diabetes [19,20], and insulin pump therapy has a similar impact (though more loosely associated than CGM) [21,22,23,24]. Automated insulin delivery systems have been found to not only improve HbA1c, but also to improve the percentage of time glucose is in the target range and may reduce events of hyperglycemia and hypoglycemia when compared with sensor-augmented insulin pump therapy [25,26,27].

In one of the largest long-term studies on post-TPIAT outcomes by Bellin et al. reported an overall decline in islet function over time, with 13% of their cohort of patients being insulin independent at 10-years post-TPIAT and 47% with evidence of beta cell function [17]. Treatment strategies utilizing advancing diabetes technology will certainly continue to be a component of studies assessing improvements to graft function over time. In our group, the overall favoring of continuation of insulin pump therapy amongst those individuals still requiring exogenous insulin suggests acceptability of the insulin delivery method beyond the initial weeks following surgery. Our study contributes preliminary data into the exploration of the impact of diabetes technology on metabolic outcomes after TPIAT at our center. Future studies are needed to explore the impact of advancing diabetes technology in post-pancreatectomy management, as it relates to not only metabolic outcomes but also to the impact on quality of life over time.

Limitations of the study include the single center design. Assessment of outcomes was likely limited by sample size. Due to the relatively low prevalence of CP and ARP in children and adolescents who require TPIAT, it takes years to reach a sample size of statistical significance.

## 5. Conclusions

This report suggests that there are long-term benefits from early pump use compared to MDI post-TPIAT. At one-year post-TPIAT, early pump users were more likely to have better graph function, be insulin independent, and to have weaned off insulin within the first six months after surgery. Younger age at time of TPIAT and treatment group were significantly associated with insulin independence at one year. More studies are needed to investigate immediate post-operative insulin management on long-term outcomes post-TPIAT.

## Figures and Tables

**Table 1:** Insulin treatment duration.

	MDI Users n = 15	Pump Users n = 25	*p*-Value
**ICU duration (days)**	10.0 (9.0–12.0)	7.0 (7.0–8.0)	<0.0001
**IV insulin treatment duration (days)**	9.0 (8.0–11.0)	7.0 (7.0–8.0)	0.001
**SQ insulin duration (days)**	5.0 (4.0–9.0)		-

Data presented as median (25th–75th percentile).

**Table 2:** Demographic characteristics.

	All n = 40	MDI Users n = 15	Pump Users n = 25	*p*-Value *
TPIAT age (years)	13.1 (8.4–15.9)	12.4 (8.9–14.8)	13.34 (7.4–16.5)	0.48
Sex (female)	27 (68%)	10 (67%)	17 (68%)	1.00
Diagnosis				1.00
ARP	3 (7.5%)	1 (7%)	2 (8%)	
CP	37 (92.5%)	14 (93%)	23 (92%)	
BMI percentile	70.4 (44.3–93.2)	84.0 (63.2–90.3)	66.8 (42.3–93.4)	0.45
IEQ/kg	6328 (4298–8346)	6485 (5659–8441)	6211 (3462–7999)	0.34
Genetic testing positive	30 (75%)	13 (87%)	17 (68%)	0.27
PRSS1	11/39 (28%)	4/14 (31%)	7/25 (27%)	
SPINK1	10/38 (26%)	4/14 (31%)	6/24 (24%)	
CFTR	14/38 (37%)	5/14 (38%)	9/24 (36%)	
CTRC	0/33 (0%)	0/11 (0%)	0/22 (0%)	
CPA1	2/21 (10%)	1/5 (20%)	1/16 (6%)	
Islet Antibodies (+IAA)	1/38 (3%)	0/13 (0%)	1 (4%)	1.00
Sweat Chloride test (CF)				0.58
Negative	25/30 (83%)	9/10 (90%)	16/20 (80%)	
Indeterminate (30–60)	3/30 (10%)	0/10 (0%)	3/20 (15%)	
Positive (>60)	2/30 (7%)	1/10 (10%)	1/20 (5%)	
Endocrine insufficiency (impaired fasting glucose/insulin resistance)	4 (10%)	2 (13%)	2 (8%)	0.62

Data presented as median (25th–75th percentile) or n (%); * *p*-values only for tests run between two groups.

**Table 3:** Metabolic characteristics at baseline pre-TPIAT.

	MDI Users n = 15	Pump Users n = 25	*p*-Value
HbA1c %	5.2 (4.8–5.4)	5.1 (4.9–5.4)	0.89
Fasting glucose (mg/dL)	89.0 (86.0–95.0)	91.0 (84.0–93.0)	0.75
Fasting C-peptide (ng/mL)	1.3 (0.6–2.1)	1.3 (0.8–2.0)	0.87
Peak C-peptide (ng/mL)	5.0 (1.9–7.6)	4.1 (3.0–6.5)	0.93
AUC C-peptide (ng/mL/min)	303.0 (172.5–367.5) n = 13	268.5 (148.5–374.3) n = 20	1.00
AUC insulin (uIU/mL/min)	2876 (2082–4916) n = 13	1688 (1160–4904) n = 21	0.52
Insulinogenic Index	1.3 (0.5–3.2) n = 11	2.0 (0.9–3.7) n = 20	0.35
C-peptide Secretion Index	0.09 (0.04–0.15) n = 11	0.12 (0.09–0.22) n = 20	0.14
BMI Percentile	84.0 (63.2–90.3)	66.8 (42.3–93.4)	0.45

Data presented as median (25th–75th percentile).

**Table 4:** Metabolic characteristics at 12-months post-TPIAT.

	MDI Users n = 15	Pump Users n = 25	*p*-Value
HbA1c %	6.7 (5.9–7.8)	6.2 (5.6–7.4) n = 22	0.40
Fasting glucose (mg/dL)	122.0 (101.0–163.0) n = 14	97.0 (87.0–110.0) n = 21	0.003
Fasting C-peptide (ng/mL)	0.8 (0.5–1.0) n = 14	0.7 (0.5–0.8) n = 21	0.71
Peak C-peptide (ng/mL)	1.8 (1.2–2.2) n = 14	1.9 (1.6–2.4) n = 21	0.39
AUC C-peptide (ng/mL/min)	50.3 (27.0–76.5) n = 12	57.0 (42.0–118.5) n = 21	0.14
AUC insulin (uIU/mL/min)	1104 (476–1227) n = 11	1029 (611–1332) n = 19	0.67
Insulinogenic Index	0.16 (0.04–0.58) n = 14	0.19 (0.05–0.42) n = 21	0.91
C-peptide Secretion Index	0.01 (0.00–0.01) n = 14	0.01 (0.00–0.02) n = 21	0.72
BMI percentile	71.1 (59.7–93.6)	63.5 (24.3–77.8) n = 24	0.07

Data presented as median (25th–75th percentile).

**Table 5:** Modified Igls 2.0 at 12-months post-TPIAT.

β-Cell Graft Functional Status	HbA1c %	Severe Hypoglycemia	Insulin Requirements	C-Peptide (ng/mL)	Treatment Success	MDI Users n = 15	Pump Users n = 25
Optimal	≤ 6.5	None	None	Detected (>0.5)	Yes	2/14 (14%)	10/21 (48%)
Good	< 7.0	None	Yes	Detected (>0.5)	Yes	3/14 (21%)	5/21 (24%)
Marginal	≥ 7.0	None	Yes	Detected (>0.5)	No	6/14 (43%)	6/21 (29%)
Failure	≥ 7.0	None	Yes	Undetected	No	3/14 (21%)	0/21 (0%)

n = 3 patients in the pump group were lost to follow-up and n = 1 did not have c-peptide data at one year, n = 1 in the MDI group did not have c-peptide data at one year.

**Table 6:** BETA-2 score at 12-months post-TPIAT.

	MDI Users n = 14	Early Pump Users n = 21	*p*-Value
BETA-2 Score	8.0 (5.1–11.5)	13.7 (10.7–16.5)	0.06

Data presented as median (25th–75th percentile).

**Table 7:** Insulin requirement at 12-months post-TPIAT.

Insulin Requirement	All n = 40	MDI Users n = 15	Pump Users n = 25	*p*-Value
Off	12/37 (32%)	2 (13%)	10/22 (45%)	0.07 *
On	25/37 (68%)	13 (87%)	12/22 (55%)	
<0.5 TDD/kg	19/37 (51%)	9 (60%)	10/22 (45%)	
≥0.5 TDD/kg	6/37 (16%)	4 (27%)	2/22 (9%)	

* *p*-value for testing proportions on vs. off insulin. TDD/kg sub-groups are for informational purposes.

## Data Availability

The data presented in this study are available upon request from the corresponding author. The data are not publicly available due to privacy.
